# Near-Complete Genome Sequence of *Clostridium paradoxum* Strain JW-YL-7

**DOI:** 10.1128/genomeA.00229-16

**Published:** 2016-05-05

**Authors:** W. Andrew Lancaster, Sagar M. Utturkar, Farris L. Poole, Dawn M. Klingeman, Dwayne A. Elias, Michael W. W. Adams, Steven D. Brown

**Affiliations:** a Department of Biochemistry and Molecular Biology, University of Georgia, Athens, Georgia, USA; b Graduate School of Genome Science and Technology, University of Tennessee, Knoxville, Tennessee, USA; c Biosciences Division, Oak Ridge National Laboratory, Oak Ridge, Tennessee, USA

## Abstract

*Clostridium paradoxum* strain JW-YL-7 is a moderately thermophilic anaerobic alkaliphile isolated from the municipal sewage treatment plant in Athens, GA. We report the near-complete genome sequence of *C. paradoxum* strain JW-YL-7 obtained by using PacBio DNA sequencing and Pilon for sequence assembly refinement with Illumina data.

## GENOME ANNOUNCEMENT

The anaerobic thermoalkaliphile *Clostridium paradoxum* strain JW-YL-7 was isolated from an aeration pool at the municipal sewage treatment plant in Athens, GA, USA, in 1993 (1), and representatives have been identified in sewage sludge from North America, Europe, and New Zealand (2).This organism grows optimally at 56°C at a pH of 10.1, with a doubling time of 16 min; produces acetate, H_2_, and CO_2_ as major products of fermentation; and has the unusual property of highly motile sporulated cells (1). It has been proposed that *C. paradoxum* be assigned to the genus *Peptoclostridium* in the family *Peptostreptococcacae*, along with the pathogens *Clostridium difficile* and *Clostridium sordellii* and the alkalithermophilic *Clostridium thermoalcaliphilum* (3). *C. paradoxum*, unlike its neutrophilic relatives, must maintain an inverted pH gradient, with the interior of the cell more acidic than the external environment. Previous studies have investigated the structure and function of the Na^+^ translocating F_1_F_0_ ATPase of *C. paradoxum*, which functions strictly as an Na^+^ exporter to establish an electrochemical gradient to drive cellular processes such as solute import and cell motility (4, 5).

Raw PacBio RS-II data (three single-molecule real-time [SMRT] cells) were filtered as described previously (6) to generate 140,177 reads with an average length of 6,028 bp and ~310× genome coverage. PacBio reads were assembled into 3 large contigs using the HGAP.3 protocol from SMRT Analysis software version 2.2 for a genome size of 1.9 Mb and a largest contig of 1.5 Mb. Assembly polishing and refinement were performed using Illumina MiSeq reads and pilon software (7). Genome annotation was performed as described previously (8), which predicted 1,941 protein-coding genes and G+C content of 30.2%. Genome integrity was further verified by the CheckM tool (9), which classified the genome as belonging to class *Clostridia* with 98.6% completeness estimation based on the presence of 250 marker genes. Although the genome encodes a large number of genes with the greatest homology to those of thermoalkaliphiles, the species with the greatest number of genes with closest homologs to *C. paradoxum* are *C. difficile*, followed by *Clostridium sticklandii*. In the CAAAA**A** motif identified in *C. difficile*, adenine residues (shown in bold type) associated with CTGC**A**G, WC**A**GC, and CT**A**NNNNNNTAG motifs are also methylated in *C. paradoxum* (10).

The 1.9-Mb genome is less than half the size of sequenced sporulating relatives such as *C. difficile* and significantly smaller than the nonsporulating *C. sticklandii* and is not significantly larger than noncultivable spore formers of “*Candidatus* Arthromitus” species (11, 12). The nonpathogenicity, ease of cultivation, streamlined genome, and high efficiency of sporulation make *C. paradoxum* a valuable model for investigating sporulation in *Clostridia*. The previously described F-type ATPase, as well as a putative membrane-bound sodium translocating pyrophosphatase, a ferredoxin-NAD^+^:oxidoreductase (Rnf) complex possibly involved in sodium efflux and symporters of the sodium:alanine, sodium:dicarboxylate, and sodium:neurotransmitter families are present. In addition to the bifurcating Rnf complex, *C. paradoxum* possesses three soluble bifurcating hydrogenases. These genome sequence results agree with earlier analysis results indicating multiple copies of the 16S rRNA genes with heterogeneous intervening sequences (13) and will be useful for a range of studies.

### Nucleotide sequence accession numbers.

This whole-genome shotgun project has been deposited at DDBJ/ENA/GenBank under the accession LSFY00000000. The version described in this paper is version LSFY01000000.
